# Comparison of the TEMPO Binocular Perimeter and Humphrey Field Analyzer

**DOI:** 10.21203/rs.3.rs-3283528/v1

**Published:** 2023-08-29

**Authors:** Takashi Nishida, robert WEINREB, Juan Arias, Cristiana Vasile, Sasan Moghimi

**Affiliations:** Hamilton Glaucoma Center, Shiley Eye Institute, University of California San Diego; Hamilton Glaucoma Center, Shiley Eye Institute, University of California San Diego; Hamilton Glaucoma Center, Shiley Eye Institute, University of California San Diego; Hamilton Glaucoma Center, Shiley Eye Institute, University of California San Diego; Hamilton Glaucoma Center, Shiley Eye Institute, University of California San Diego

## Abstract

This study compared between TEMPO, a new binocular perimeter, with the Humphrey Field Analyzer (HFA). Patients were tested with both TEMPO 24 − 2 AIZE-Rapid and HFA 24 − 2 SITA-Fast in a randomized sequence on the same day. Using a mixed-effects model, visual field (VF) parameters and reliability indices were compared. Retinal nerve fiber layer (RNFL) thickness was measured using Cirrus OCT, and coefficient of determinations for visual field and OCT parameters were calculated and compared using Akaike information criteria. 740 eyes (including 68 healthy, 262 glaucoma suspects, and 410 glaucoma) of 370 participants were evaluated. No significant differences were seen in mean deviation and visual field index between the two perimeters (P > 0.05). A stronger association between VF mean deviation and circumpapillary RNFL was found for TEMPO (adjusted R^2^ = 0.28; AIC = 5210.9) compared to HFA (adjusted R^2^ = 0.26; AIC = 5232.0). TEMPO had better reliability indices (fixation loss, false positive, and false negative) compared to HFA (all P < 0.05). Measurement time was faster for TEMPO compared to HFA (261sec vs. 429sec, P < 0.001). Further investigations are needed to assess the long-term monitoring potential of this binocular VF test.

## Introduction

Glaucoma is an optic neuropathy characterized by the gradual loss of retinal ganglion cells and their axons, which can lead to vision loss.^1^ Clinical detection and monitoring of glaucoma involves the assessment of functional vision loss using visual field (VF) testing, and also measuring structural loss through optical coherence tomography (OCT).^2^ VF testing demands active participation from patients and presents several challenges such as lengthy test durations and high variability due to its subjective nature.^3^ TEMPO, formally called IMOvifa, is a novel standard automated perimeter with binocular random testing.^4–6^ Recent studies have suggested that binocular VF testing may effectively suppress eye movements and stabilize fixation, thus potentially enhancing the reliability of test results.^7^ Moreover, this device also adjusts the stimulus presentation point by tracking eye movements.^6,8,9^

We hypothesized that this new technology could reduce testing duration and patient fatigue, minimize variability in test results, and improve the correlation between structural and functional data. The purpose of this study was to compare the TEMPO with the Humphrey visual field analyzer (HFA), the most widely used automated perimeter.

## Results

740 eyes (including 68 healthy, 262 glaucoma suspects, and 410 glaucoma) of 370 participants (mean age, 67.6 years [95% CI: 66.6–68.6]; 222 female [60.0%] and 148 male [40.0%]; and 219 White [59.2%], 58 Asian [15.7%], 20 African American [5.4%], 44 other or mixed race [11.9%], and 28 unknown or not reported race [7.6%]) were included. Demographic and baseline clinical characteristics of the participants are presented in Table 1.

The comparison for VF parameters and reliability indices between HFA and TEMPO is summarized in Table 2. No significant differences were seen in MD and VFI between the two perimeters (P>0.05). While significant differences were seen in PSD (4.1 [3.9, 4.4] dB for HFA and 4.7 [4.5, 5.0] dB for TEMPO; P<0.001) and foveal threshold (33.3 [32.9, 33.7] dB for HFA and 30.8 [30.2, 31.3] dB for TEMPO; P<0.001). Bland−Altman scatterplots showed reasonable agreement between the two perimeters. The mean difference (95% LoA) was −0.2 (−4.8, 4.3) dB for MD, −0.6 (−4.7, 3.4) dB for PSD, and 0.4 (−12.7, 13.4) for VFI, respectively (Supplemental Figure 1). TEMPO had better reliability indices (Figure 2); fixation loss (11.2 [10.0, 12.5] % for HFA and 8.9 [7.3, 10.5] % for TEMPO; P=0.017), false positive (4.1 [3.7, 4.6] % for HFA and 1.3 [1.1, 1.5] % for IMOvifa; P=0.017), and false negative (4.3 [3.8, 4.8] % for HFA and 0.4 [0.3, 0.4] % for TEMPO; P<0.001). Measurement time was faster for TEMPO AIZE-Rapid compared to HFA SIFA-Fast (261 sec vs. 429 sec; P<0.001).

A stronger association between VF mean deviation and circumpapillary RNFL was found for TEMPO (adjusted R^2^ = 0.28; AIC = 5210.9) compared to HFA (adjusted R^2^ = 0.26; AIC = 5232.0). Moreover, TEMPO demonstrated higher structure-function relationships compared to HFA in all quadrants (Table 3). The inferior quadrant for RNFL had the highest association, followed by superior, temporal, and nasal quadrant for RNFL (adjusted R^2^ = 0.31, 0.29, 0.15, and 0.06 for TEMPO, respectively).

Supplemental Figure 2 provides a summary of the usability findings. 73% of participants preferred TEMPO, while 17% preferred HFA. 83% of participants reported no difficulties with TEMPO. Furthermore, TEMPO received positive feedback in terms of screen readability, ease of concentration, and shorter test duration, as compared to HFA.

## Discussion

In this study, we prospectively performed VF testing with both TEMPO AIZE-Rapid and HFA SITA-Fast in a randomized order and identified a stronger structure-function relationship and better reliability indices with TEMPO compared to HFA. TEMPO reduced measurement time by approximately 40% without compromising perimetric performance. Even though the participants were inexperienced with TEMPO prior to the study, it was strongly preferred by patients.

Effective glaucoma management necessitates functional and structural exams, and correlating these changes ensures reliable tracking of disease progression.^10,11^ Clinicians should optimize and balance considerations such as medical burden, patient preferences, and efficient detection of disease progression to prevent lifelong visual loss. Previous studies have shown that using a combination of structural and functional analyses enhances the ability to detect glaucoma and its progression.^12–14^ The current cross-sectional study shows that TEMPO had a stronger structure-function relationship with Cirrus OCT compared to HFA, both globally and sectorally. Our findings support the study by Bowd et al., investigating structure-function relationships using Stratus OCT. They found stronger associations (R^2^ = 0.33–0.38) in the inferotemporal disc, followed by modest associations in the superotemporal disc area (R^2^ = 0.19–0.25), and weak associations in the temporal disc area (R^2^ = 0.02–0.03).^15^ The lower structure-function relationship in the temporal quadrants, compared to the superior/inferior quadrants, may be attributed to two factors: the higher variability caused by relatively fewer measurement points for visual field and the position of the optic nerve head in relation to the fovea.^16^ Individual anatomical differences, such as variations in the shape, rotation, and tilt of the ONH, can affect the results of structure-function relationship.^10^ Both VF and OCT measurements are prone to inter-subject and test-retest variability, which are major causes of discrepancy in structure-function relationship.^10,17^ We speculate that due to its reduced fatigue and higher reliability of TEMPO lead to reduced variability. However, both short-term and long-term reproducibility need to be confirmed in future studies.

HFA SITA-Fast and TEMPO AIZE-Rapid have several items in common regarding reliability indices, but there are some differences. First, false positives are calculated by both devices using reaction time. HFA SITA-Fast uses the percentage of stimuli responded to within a minimum reaction time with an adjustment for the average reaction time of the individual patient.^18^ In contrast, TEMPO AIZE-Rapid uses the percentage of those that have a reaction time of less than 0.3 seconds. Second, false negatives are calculated as percentage of not responding by presenting a 9 dB bright target to the determined threshold in HFA SITA-Fast, while TEMPO AIZE-Rapid uses percentage of not responding by presenting ≥ 2 dB bright target in the process of threshold determination for all stimuli. Third, fixation loss is calculated as percentage in response to stimulus to blind spots, which is known as Heijl Krakau method, in HFA SITA-Fast. In contrast, TEMPO AIZE-Rapid uses the percentage of stimuli with Gaze tracking greater than 5 degrees. Although the Heijl-Krakau method was introduced in the 1970s and is considered the gold standard,^19^ it has several disadvantages that include time-consuming catch trials, infrequent fixation check, and inaccuracy of fixation loss ratio when the blind spot location is dislocated. In principle, gaze tracking, which monitors the movement of the pupils, should be expected to measure fixation monitoring more accurately, and also is used in HFA SITA-Faster.^20^ In the current study, TEMPO AIZE-Rapid demonstrated lower values than HFA SITA-Fast for all reliability indices. This may be related to the reduction of patient fatigue due to shorter examination time and the difference in approach of non-occlusion. The manufacturer-recommended limits flagged 17.1% of HFA and 10.6% of TEMPO results as low reliability. Fixation losses were the main cause of low reliability for both perimeters (14.4% for HFA and 8.2% for TEMPO). Previous studies have also identified fixation losses as the primary cause of unreliable VF classifications.^21,22^ Comparing these numbers is challenging due to methodological differences, but incorporating more accurate and reliable techniques into future device, while maintaining efficacy, is crucial.

This study has several limitations. First, it was based on participants from a tertiary care academic practice, which could introduce certain biases in socio-economic status, demographics, and severity of disease, which could differ from those of patients treated in other settings. This could potentially restrict the generalizability of our findings. Second, the current software is derived from a database that only consists of data from the Japanese population. Regardless of this limitation, HFA and TEMPO exhibited excellent agreement. Third, certain situations, such as conditions of binocular vision dysfunction like strabismus, anisometropia, nystagmus among others, can render binocular open-eye examinations unfeasible.^23^ These conditions were not evaluated in our study. Last, all participants were using TEMPO for the first time, while they had previous experience with HFA. Although learning effects tend to increase the false positive rate for inexperienced examinees,^24^ false positives were relatively low for TEMPO in our study.

In conclusion, TEMPO showed a stronger structure-function relationship with Cirrus OCT. It also showed better reliability compared to the HFA. Further studies are necessary to evaluate the potential of this binocular VF test for longitudinal monitoring.

## Methods

### Participants

Participants were recruited from patients at the Shiley Eye Institute, University of California, San Diego. The research protocol followed the tenets of the Declaration of Helsinki and was approved by the University of California, San Diego Institutional Review Board. All study participants provided written informed consent.

The participants’ eyes were divided into three diagnostic groups: healthy, glaucoma suspect, and glaucoma. Healthy eyes were characterized by IOP ≤ 21 mmHg, normal-appearing optic discs and neuroretinal rims, and normal VF test results defined as pattern standard deviation (PSD) within the 95% CI and Glaucoma Hemifield Test (GHT) results within normal limits using Swedish Interactive Threshold Algorithm (SITA) 24 − 2 FAST. Glaucoma suspects were defined as eyes with IOP of ≥ 22mmHg or glaucomatous-appearing optic discs (glaucomatous optic neuropathy) without repeatable glaucomatous VF damage. Glaucomatous optic neuropathy was defined as excavation, the presence of focal thinning, notching of the neuroretinal rim, or localized or diffuse atrophy of the RNFL by attending physicians based on ophthalmoscopic examination or fundus photographs. Glaucoma was defined as eyes showing at least two reliable (fixation losses and false negatives ≤ 33% and ≤ 15% false positives) and repeatable abnormal (GHT outside normal limits or PSD outside 95% normal limits) VF results using the 24 − 2 SITA-FAST with similar glaucomatous defect patterns on consecutive testing as evaluated by study investigators. Glaucoma included all types, such as primary open-angle glaucoma, primary angle closure glaucoma, and secondary glaucoma. Eyes were excluded if they had any other ocular or systemic conditions, apart from glaucoma, that could affect VF test results, such as age-related macular degeneration.

### Visual Field Testing

TEMPO (CREWT Medical Systems, Tokyo, Japan) is the improved successor to the portable head-mounted perimeter device known as imo.^8^ This device has two optical systems and pupil-monitoring systems for each eye, allowing independent target presentations and pupil monitoring.^8^ It enables separate testing of each eye and can randomly present test indicators to either eye, with both eyes open, without the examinee knowing which eye is being tested (binocular random single-eye test). AIZE employs Bayesian inference and maximum likelihood methods to determine the threshold, and reduces test time by around 70% compared to the 4 – 2 dB bracketing method.^8^ AIZE-Rapid maintains the AIZE test method but enhances the representation of interaction with adjacent measurement points.^4^

For the current study, all patients underwent HFA 24 – 2 SITA-Fast and TEMPO 24 – 2 Ambient Interactive Zippy Estimated by Sequential Testing (AIZE)-Rapid on the same day in a randomized order using Goldmann size III (0.431° visual angle) stimuli. Since the purpose of this study was to compare HFA and TEMPO, no exclusions were made at specific cutoff values for reliability indices (fixation losses, false negatives and false positives) for both devices. The binocular random testing mode was selected for testing with TEMPO. In addition, to evaluate usability for patients, there was a questionnaire, as follows: 1) Which device do you prefer?, 2) Did you have any difficulty with the simultaneous examination of both eyes using the novel device?, 3) Was the screen easy to see?, 4) Was it easy to concentrate?, 5) Was the test time short?. These questions were assessed using a 5-point Likert scale.

### Structure-function relationship

**Figure 1:**
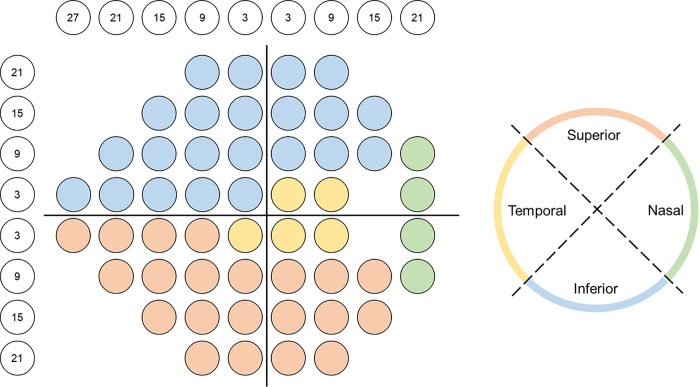
A topographic map relating visual field test stimulus locations (left) to OCT-measured retinal nerve fiber layer thickness (right) is shown for a right eye.

**Figure 2:**
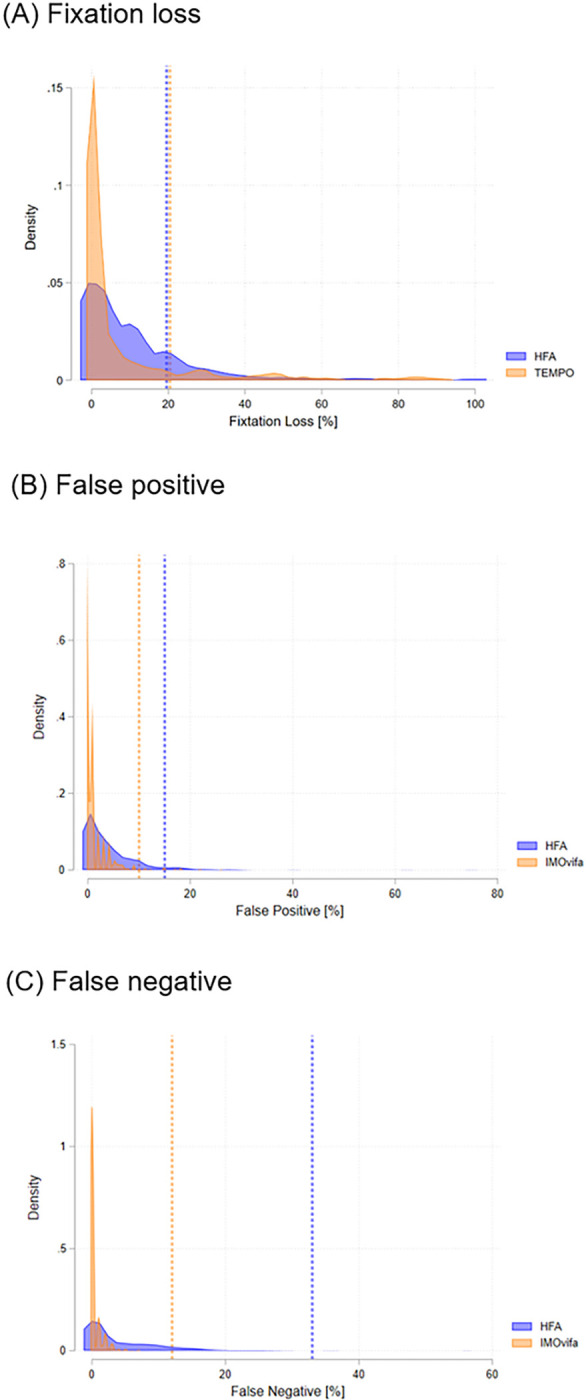
Comparison of reliability indices between Humphrey Field Analyzer and TEMPO. Density plot represents the distribution of (A) fixation losses, (B) false positives, and (C) false negatives. Vertical lines indicate the manufacturer recommended reliable cutoff values.

Retinal nerve fiber layer thickness (RNFL) was measured using Cirrus spectrum-domain OCT (Carl Zeiss Meditec, Inc, Dublin, CA) Optic Disc Cube 200×200 protocol scans. A 3.46 mm diameter circle was automatically placed around the optic disc, providing RNFL thickness globally and in superior, inferior, temporal, and nasal sectors. Coefficient of determination for VF and OCT parameters was calculated and compared using Akaike information criteria (AIC). Age adjusted R^2^ values and AIC were used to compare the models for goodness of fit. The higher R^2^ and smaller AIC mean better fit. To compare the strength of structure-function relationship between HFA and TEMPO, the absolute value of the residuals from each model were calculated and compared using mixed effects model.^15^ Structure-function relationships were investigated for global (VF mean deviation [MD] and circumpapillary RNFL) and sectoral parameters (VF mean sensitivity and quadrant RNFL) based on simplified map proposed by Garway-Heath et al. (Fig. 1).^26,27^ Mean sensitivity was calculated in dB by converting the threshold sensitivity of each test point to a linear scale and then averaging them to obtain the mean sensitivity values.

## Statistical analysis

Patient and eye characteristics were reported as mean (95% CI) for continuous data and count (percentage) for categorical data. MD, pattern standard deviation (PSD), foveal threshold (FT), and visual field index (VFI), and reliability indices (fixation loss, false positive, and false negative) were compared using mixed-effects model between the two perimeters. Reliability indices were illustrated in a kernel density estimate plot to compare the values from two perimeters. Kernel density estimate is a non-parametric way to estimate the probability density function of a random variable. In other words, it provides a smoothed version of a histogram, giving a continuous curve. The Bland-Altmann plot assessed the limits of agreement (LoA) between the two perimeters. Measurement time for performing VF for both eyes was recorded for each device. It only accounted for the actual examination time and excluded the setup time needed for testing the second eye with HFA. All statistical analyses were performed with Stata software (version 15; StataCorp, College Station, Texas). Statistical significance for tests was set at P ≤ 0.05.

## Figures and Tables

**Table 1.** Demographic and Baseline Clinical Characteristics of the Participants

Characteristic	n=740 eyes of 370 participants

Age (years)	67.6 (66.6, 68.6)

Sex (% Female)	222 (60.0%)

Race, n (%)	
White	219 (59.2%)
Asian	58 (15.7%)
African American	20 (5.4%)
Other or mixed race	44 (11.9%)
Unknown or not reported	28 (7.6%)

Ethnicity, n (%)	
Non-Hispanic	308 (83.2%)
Hispanic	31 (8.4%)
Multi-racial	6 (1.6%)
Unknown or not reported	25 (6.8%)

Diagnosis, n (%)	
Healthy	68 (9.2%)
Glaucoma suspects	262 (35.4%)
Glaucoma	410 (55.4%)

Disease Severity by HFA 24–2 VF MD, Eye No. (%)	
Early glaucoma (VF MD>-6),	250 (61.0%)
Moderate and advanced glaucoma (VF MD≤-6)	160 (39.0%)

MD = mean deviation; VF = visual field. Values are shown in mean (95% confidence interval), unless otherwise indicated.

**Table 2.** Comparison between Humphrey Field Analyzer and IMOvifa

	TEMPO	Humphrey Field Analyzer	Mean deference	P value^a^
Mean deviation, dB	−4.0 (−4.5, −3.6)	−4.3 (−4.7, −3.9)	−0.2 (−0.7, 0.2)	0.283
Pattern standard deviation, dB	4.7 (4.5, 5.0)	4.1 (3.9, 4.4)	−0.6 (−0.9, −0.4)	**<0.001**
Visual field index, %	88.9 (87.6, 90.2)	89.2 (88.0, 90.4)	0.4 (−1.0, 1.7)	0.591
Fixation loss, %	8.9 (7.3, 10.5)	11.2 (10.0, 12.5)	−2.1 (−3.9, −0.4)	0.017
False positive, %	1.3 (1.1, 1.5)	4.1 (3.7, 4.6)	−2.8 (−3.2, −2.4)	**<0.001**
False negative, %	0.4 (0.3, 0.4)	4.3 (3.8, 4.8)	−3.9 (−4.4, −3.5)	**<0.001**
Measurement time for both eyes, sec	260.6 (256.2, 265.0)	429.1 (422.0, 436.1)	−168.5 (−172.4, −164.5)	**<0.001**

Values are shown in mean (95% confidence interval), unless otherwise indicated.

a Mixed-effects model

**Table 3.** Comparison of Topographic Structure-functional Relationship between Humphrey Field Analyzer and TEMPO

Variable	Humphrey Field Analyzer		TEMPO		P-value
	Adjusted-R^2^	AIC	Adjusted-R^2^	AIC	
Circumpapillary RNFL and mean deviation	0.26	5232.0	0.28	5210.9	<0.001
Inferior RNFL and superior MS	0.30	3958.7	0.31	3930.1	<0.001
Superior RNFL and inferior MS	0.28	3510.7	0.29	3497.2	<0.001
Temporal RNFL and temporal MS	0.10	3511.6	0.15	3268.1	<0.001
Nasal RNFL and nasal MS	0.06	3725.1	0.06	3463.0	<0.001

AIC = Akaike information criteria; RNFL = retinal nerve fiber layer; MS = mean sensitivity. Age was adjusted in all models.

## Data Availability

The datasets used and/or analysed during the current study available from the corresponding author on reasonable request.
